# Muscle Mass Is a Better Predictor of Survival in Dogs With Chronic Kidney Disease Compared to Body Condition Score

**DOI:** 10.1111/jpn.70061

**Published:** 2026-04-22

**Authors:** Thiago Henrique Annibale Vendramini, Pedro Henrique Marchi, Vivian Pedrinelli, Andressa Rodrigues Amaral, Laís Oyama Cotrim Lima, Bianca Petermann Moretti, Natália Manuela Cardoso de Oliveira, Cristiana F. F. Pontieri, Juliana T. Jeremias, Júlio Cesar de Carvalho Balieiro, Marcio Antonio Brunetto

**Affiliations:** ^1^ Pet Nutrology Research Center, Department of Animal Nutrition and Production, School of Veterinary Medicine and Animal Science University of São Paulo Pirassununga São Paulo Brazil; ^2^ Veterinary Nutrology Service, Veterinary Teaching Hospital, School of Veterinary Medicine and Animal Science University of São Paulo São Paulo Brazil; ^3^ Nutritional Development Center of PremieRpet Grandfood Industria e Comercio LTDA Dourado São Paulo Brazil

**Keywords:** body condition score, chronic kidney disease, muscle mass score, survival rate

## Abstract

Monitoring prognostic factors, such as body weight, is important to assess the effectiveness of therapies in patients with chronic kidney disease (CKD) and estimate the survival rate (SR). As established in the literature, both body condition score (BCS) and muscle mass score (MMS), when analysed individually, show a positive correlation with SR in dogs with CKD. However, no studies have analysed the correlation between both parameters on SR. The present study aimed to clarify the real influence of fat and lean mass on the SR in dogs with CKD, based on a Cox proportional hazards regression analysis, to improve the nutritional management of these patients. This study included 120 client‐owned adult dogs diagnosed with CKD stages 2, 3, or 4. The hazard ratios for stage, BCS, MMS, age, and sex were estimated in univariate and multivariable analyses. The results showed survival was not influenced by BCS alone but was influenced by MMS. Severe muscle mass loss (MMS 0) had a 3.85‐fold risk of mortality when compared to normal muscle mass (*p* = 0.006). Regardless of body condition, the present study observed that what really influenced the survival of dogs with CKD, considering the stage, was muscle mass.

## 1 Introduction

Defined as a progressive and irreversible loss of nephrons persisting for at least 3 months, chronic kidney disease (CKD) is considered the most common kidney disease in dogs (Polzin 2011a). The prevalence of this condition may vary, with results fluctuating between 0.5% and 3.0% in the general population and around 10% in the hospitalized canine population (Polzin 2011b; Vertloo 2025). Furthermore, according to the International Renal Interest Society (IRIS), the estimated prevalence of CKD in the general canine population is between 0.5% and 1.0%, but it increases with age (Roura 2019). CKD may originate from congenital malformations, chronic pyelonephritis, systemic arterial hypertension, infections, or nephrotoxicity, but in general, its primary causes are not yet fully elucidated (Polzin 2011a; Polzin et al. 1983). Diagnosis is based on the detection of changes in the clinical condition, blood pressure, urinalysis, haematology, biochemical, and ultrasound analyses (Pelander et al. 2019).

The recommended treatment of CKD may vary according to the stage of the disease, as proposed by IRIS (International Renal Interest Society 2019), and aims to improve both quality of life and life span (Bartges 2012; Polzin 2011a). In addition, from a nutritional perspective, monitoring body condition score (BCS) and muscle mass score (MMS) is important, since both are positively correlated with the survival rate (SR) in dogs and can be used as prognostic parameters (Parker and Freeman 2011; Pedrinelli et al. 2020; Rudinsky et al. 2018). However, considering the physiology of adipose tissue, the relationship between higher BCS and SR is contradictory, as excessive body fat accumulation negatively affects most physiological functions and thus is considered a risk factor for many chronic diseases, including renal disease (German et al. 2010; Tvarijonaviciute et al. 2012). This may be related to the increased expression of pro‐inflammatory adipokines by obese adipocytes, which predisposes individuals to various oxidative stresses associated with reduced anti‐inflammatory response (Bastien et al. 2015; German et al. 2010; Vendramini et al. 2020).

In human medicine, the correlation between overweight or obese patients and better prognosis in certain chronic diseases is called the obesity paradox (Gruberg et al. 2002). According to the currently available literature, most studies in veterinary medicine have also shown a positive correlation between BCS, MMS, and SR in dogs with some chronic diseases, including CKD (Brunetto et al. 2010; Ineson et al. 2019; Liu et al. 2012; Molina et al. 2018; Nentwig et al. 2016; Parker and Freeman 2011; Pedrinelli et al. 2020; Rudinsky et al. 2018). However, BCS is an indicator of adiposity, while MMS is an indicator of lean body mass, and the two are at least somewhat independent of each other. Thus, the objective of the present study was to analyse the influence of BCS and MMS independently on the SR in dogs with CKD, to improve the nutritional approach for these patients.

## 2 Materials and Methods

### 2.1 Data Collection

Medical records from dogs evaluated between 2013 and 2025 by the Nutrition Consultation Practice of the Internal Medicine Service of the Veterinary Teaching Hospital of the School of Veterinary Medicine and Animal Science—University of São Paulo, Brazil, were retrospectively reviewed. Data obtained from records included age, breed, sex, body weight at the time of evaluation, BCS, MMS, serum creatinine concentration, age at diagnosis, stage of CKD, and survival time, which was calculated as the interval between diagnosis and death. The date of death was obtained from hospital records and, when unavailable, through telephone contact with the owner.

Dogs were weighed, and BCS and MMS were determined by visual inspection and palpation of key anatomical regions. BCS was assessed on a scale from 1 to 9 (Laflamme 1997), and MMS was evaluated on a scale from 0 to 3 (Michel et al. 2011). The diagnosis and staging of CKD were based on urine specific gravity of 1.030 or lower and serum creatinine concentrations exceeding 1.4 mg/dL persisting for at least 3 months, as those aspects characterize individuals diagnosed with CKD stages 2 or higher (International Renal Interest Society 2019). Multiple veterinarians participated in the assessment of the dogs, including diagnosis, physical examination, and evaluation of BCS and MMS.

### 2.2 Animals

The inclusion criteria comprised client‐owned dogs older than 12 months and diagnosed with CKD stages 2, 3, or 4 (International Renal Interest Society 2019) without any comorbidities, regardless of breed or sex. To include animals with higher BCS for the analyses, overweight and obesity were not considered as comorbidities. The criteria for exclusion of animals were absence of return after the first clinical evaluation; incomplete records that did not contain the required information; treatment for CKD initiated before the assessment; concomitant acute or chronic diseases (including congenital kidney disease); and pregnancy or lactation. Given these criteria, from a database of 313 dogs with CKD, 120 were included as described in Table 1.

**Table 1:** Average values of body weight, age, body condition score, and muscle mass score from the 120 dogs included in the study.

Parameters	Average ± standard deviation
Body weight (kg)	13.71 ± 11.32
Age (years)	11.51 ± 3.53
BCS^a^	4.42 ± 2.02
MMS^b^	1.73 ± 0.86
IRIS^c^ stage	2.77 ± 0.76

^a^ BCS: Body condition score.

^b^ MMS: Muscle mass score.

^c^ IRIS: International renal interest society.

### 2.3 Statistical Analyses

The SR curves were calculated according to the Kaplan‐Meier method. To identify independent prognostic factors, a Cox proportional hazards (CPH) regression analysis was performed. Univariate analyses were conducted for IRIS stage, BCS, MMS, age, and sex. As the IRIS stage variable violated the proportional hazard assumption criteria, the multivariable model was stratified by IRIS stage. The model included the variables BCS, MMS, age, and sex. Values of *p* ≤ 0.05 were considered significant.

## 3 Results

Among the 120 dogs enroled in the study, the median survival time was 128 days. The median survival times for each variable category are described in Table 2.

**Table 2:** Median survival times for each variable category.

Variable	Category	*N*	Median (days)	CI 95%
MMS^a^	MMS 3	22	246	71–1019
	MMS 2	53	143	95–310
	MMS 1	35	106	69–184
	MMS 0	10	37	3–87
BCS^b^	Obese BCS (8–9)	11	475	10–1160
	Overweight BCS (6–7)	22	263	44–410
	Ideal BCS (4–5)	37	99	76–253
	Low BCS (1–3)	50	106	66–191
IRIS^c^ Stage	Stage 4	24	13	6–65
	Stage 3	45	128	66–187
	Stage 2	51	370	206–573
Sex	Male	58	109	78–246
	Female	62	143	87–266

^a^ MMS: Muscle mass score.

^b^ BCS: Body condition score.

^c^ IRIS: International renal interest society.

**Figure 1:**
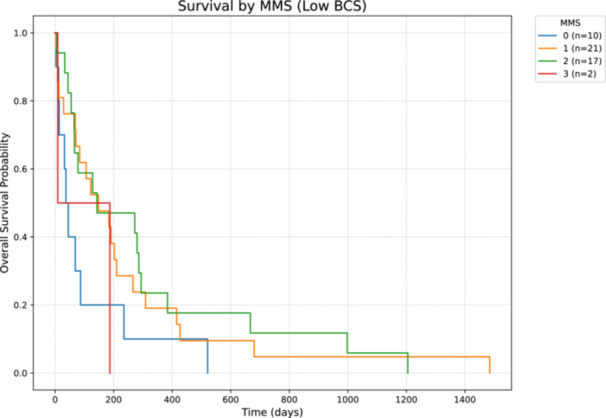
Survival rate of dogs with chronic kidney disease with low BCS (1–3). [Color figure can be viewed at wileyonlinelibrary.com]

**Figure 2:**
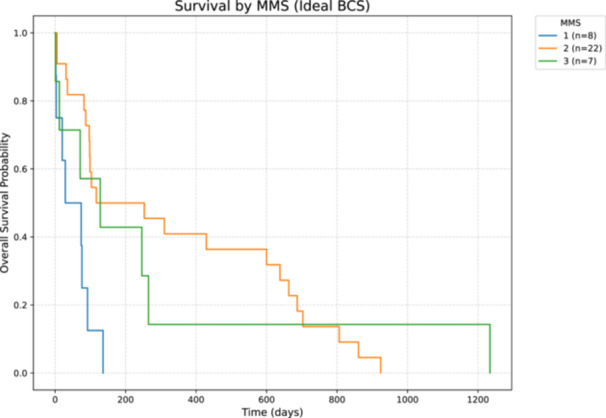
Survival rate of dogs with chronic kidney disease with ideal BCS (4–5). [Color figure can be viewed at wileyonlinelibrary.com]

**Figure 3:**
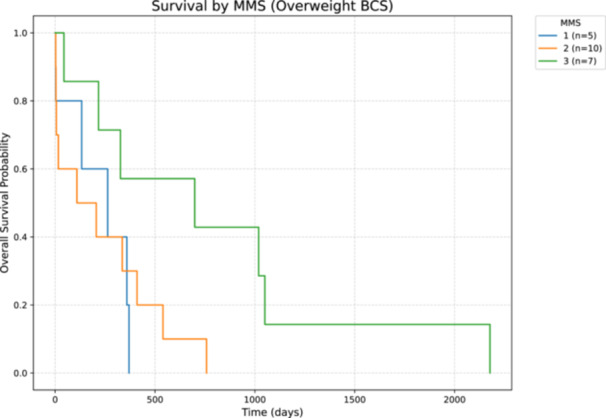
Survival rate of dogs with chronic kidney disease with overweight BCS (6–7). [Color figure can be viewed at wileyonlinelibrary.com]

**Figure 4:**
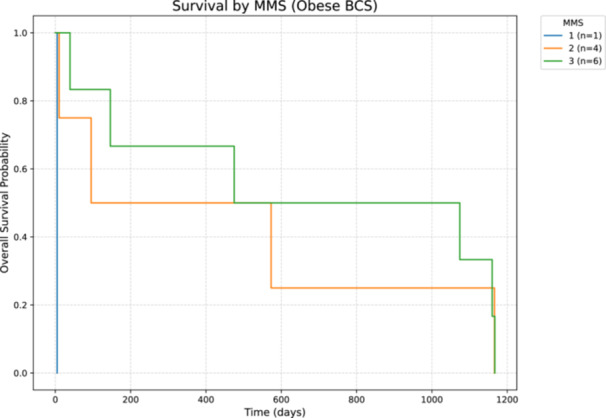
Survival rate of dogs with chronic kidney disease with obese BCS (8–9). [Color figure can be viewed at wileyonlinelibrary.com]

**Figure 5:**
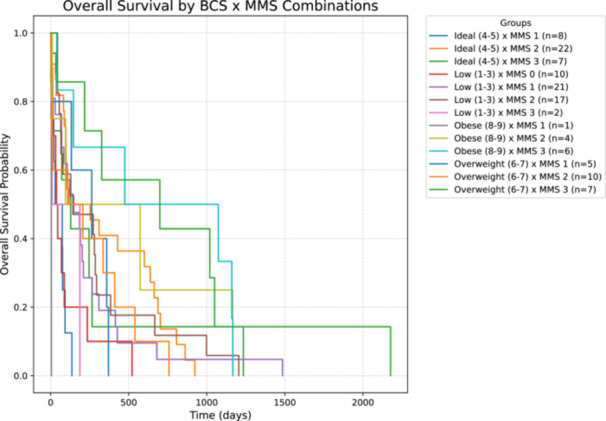
Survival rate of dogs with chronic kidney disease grouped by MMS score (0–3) and BCS (Low (1–3), Ideal (4–5), Overweight (6–7), and Obese (8–9). [Color figure can be viewed at wileyonlinelibrary.com]

For the analyses, BCS scores were grouped as follows: Low BCS (1–3), Ideal BCS (4–5), Overweight BCS (6–7), and Obese BCS (8–9), and each MMS score was analysed separately. Kaplan‐Meier curves were plotted for MMS, with different curves for each BCS group (Figures 1, 2, 3, 4) and a combination of MMS and BCS groups (Figure 5).

Cox proportional hazards regression was performed to estimate Hazard Ratios (HR) for each variable. Univariate analyses (Table 2) revealed that IRIS stage and MMS were significantly associated with survival (*p* ≤ 0.05).

The multivariable Cox model (Table 3) was stratified according to IRIS stage and identified MMS 0 as being significantly associated with reduced survival (HR = 3.85; *p* = 0.006), while MMS 1 showed a borderline association (HR = 1.97; *p* = 0.057). Age, BCS and sex had no association with survival in either univariate or multivariable analyses (Table 4).

**Table 3:** Univariate Cox proportional hazards analyses of survival of dogs with chronic kidney disease.

Variable	Category	HR	CI 95%	*p* value
MMS^a^ (reference: MMS 3)	MMS 2	1.62	0.96–2.73	0.068
	MMS 1	2.35	1.34–4.12	0.003
	MMS 0	3.88	1.77–8.51	0.001
BCS^b^ (reference: Ideal BCS [4–5])	Obese BCS (8–9)	0.57	0.29–1.14	0.111
	Overweight BCS (6–7)	0.72	0.42–1.23	0.228
	Low BCS (1–3)	1.11	0.72–1.71	0.632
IRIS^c^ Stage (reference: Stage 2)	Stage 4	4.52	2.70–7.55	< 0.001
	Stage 3	2.15	1.41–3.27	< 0.001
Age	(Per year at diagnosis)	1.01	0.96–1.06	0.793
Sex (reference: Female)	Male	0.95	0.66–1.37	0.787

^a^ MMS: Muscle mass score.

^b^ BCS: Body condition score.

^c^ IRIS: International renal interest society.

**Table 4:** Multivariable Cox proportional hazards analysis of survival of dogs with chronic kidney disease stratified by IRIS stage.

Variable	Category	HR	CI 95%	*p* value
MMS^a^ (reference: MMS 3)	MMS 2	1.31	0.71–2.39	0.3880
	MMS 1	1.97	0.98–3.96	0.057
	MMS 0	3.85	1.46–10.13	0.006
BCS^b^ (reference: Ideal BCS [4–5])	Obese BCS (8–9)	0.82	0.38–1.77	0.607
	Overweight BCS (6–7)	0.76	0.43–1.36	0.354
	Low BCS (1–3)	0.68	0.40–1.17	0.166
Age	(Per year at diagnosis)	1.01	0.95–1.07	0.801
Sex (reference: Female)	Male	0.96	0.64–1.42	0.832

^a^ MMS: Muscle mass score.

^b^ BCS: Body condition score.

## 4 Discussion

This study is the first to evaluate the specific influence of BCS and MMS on the survival of canine patients with chronic kidney disease. Although higher BCS has been previously associated with improved survival, both multivariable and univariate analyses of our data showed that MMS, rather than BCS, had a significant association with survival. Severe muscle loss (MMS 0) was associated with a 3.85‐fold increase in the risk of mortality (HR = 3.85; 95% CI = 1.46–10.13; *p* = 0.006) compared to the reference group (MMS 3), even after stratifying by IRIS stage. Regarding these outcomes, it is important to note that the mortality recorded in our cohort includes both spontaneous death and euthanasia. Severe muscle loss can contribute to poor quality of life, which is frequently a reason for owners and practitioners to consider euthanasia (Pegram et al. 2021). This, in turn, may contribute to the higher mortality observed in this group. Therefore, these results reinforce the importance of preserving muscle mass in animals with chronic kidney disease and contribute to advancing the understanding of the obesity paradox.

Progressive loss of weight and muscle mass in chronic diseases such as CKD is a recognized phenomenon in veterinary medicine. Its pathophysiology involves several dysregulated pathways that lead to an imbalance between catabolism and anabolism, independent of food intake. This process, known as cachexia, is characterized by pathological weight and/or muscle mass loss that is not reversed by nutritional intervention, and its occurrence is associated with a worse prognosis (Von Haehling et al. 2009).

Some pathways of dysregulated proteolysis associated with muscle mass loss have been described in studies linking cachexia to chronic heart disease and CKD. The most commonly reported pathway related to CKD, based on its pathophysiology, is ATP depletion. This pathway can be influenced by variations in blood pH and is most effectively activated under acidic conditions, leading to increased muscle tissue breakdown (Bailey et al. 1996).

The obesity paradox is a phenomenon described in human medicine in which individuals with a higher body mass index (BMI) affected by chronic diseases have better outcomes than those with lower BMI. The term “paradox” is used precisely because such conditions should cause greater damage, since chronic and progressive diseases are marked by altered production and secretion of hormonal and protein inflammatory mediators called adipokines (Freeman 2012; Pawelec et al. 2014). This phenomenon is also observed in overweight dogs and cats (Okada et al. 2017; Vendramini et al. 2020; Zapata et al. 2017). It should be expected that adipokines would potentiate chronic inflammation, clinical signs, and progression of chronic diseases, resulting in a shorter SR. However, literature demonstrates that high BMI is correlated with higher SR in human patients with cardiovascular diseases (Gupta et al. 2016; Horwich et al. 2001; Romero‐Corral et al. 2006), cancer (Juszczyk et al. 2020; Lennon et al. 2016), diabetes (Carnethon et al. 2012), respiratory diseases (Yamauchi et al. 2014), renal diseases (Bagheri et al. 2016; Kumakura et al. 2010; Park et al. 2014; Vashistha et al. 2014), and hospitalized patients (Isabel T. D. Correia 2003; Pirlich et al. 2006). In addition, this information is also sustained by meta‐analyses, which improve its scientific credibility (Bagheri et al. 2015; Niedziela et al. 2014; Romero‐Corral et al. 2006; Tan et al. 2016). Although there is not yet enough evidence in veterinary medicine, the obesity paradox was evidenced in studies that correlated BCS and MMS with the longevity of dogs with lymphoma (Romano et al. 2016), cardiomyopathies (Ineson et al. 2019; Slupe et al. 2008), CKD (Parker and Freeman 2011; Pedrinelli et al. 2020; Rudinsky et al. 2018), and hospitalized dogs (Brunetto et al. 2010; Liu et al. 2012; Molina et al. 2018). However, it is necessary to highlight that most of the evidence in the literature is from retrospective correlational studies, which have limitations and do not prove causation.

Although fat tissue functions as an energy reserve, which should mitigate the loss of muscle mass for energy, the pathophysiology of canine obesity is associated with higher oxidative stress and blood circulation of pro‐inflammatory adipokines such as resistin, leptin, tumour necrosis factor alpha (TNF‐α), interleukin‐1 (IL‐1), interleukin‐6 (IL‐6), interleukin‐8 (IL‐8) (Bastien et al. 2015; German et al. 2010; Tvarijonaviciute et al. 2012; Vendramini et al. 2020). These mediators appear to be related to degenerative renal injuries, such as fibrosis, apoptosis, and epithelial‐mesenchymal transdifferentiation (Nentwig et al. 2016). In CKD, the elevated production of TNF‐α, IL‐6, and IL‐8, and other mediators, induces anorexia, increases metabolic rate, and alters energy metabolism so that proteins are used as a primary source of energy, contributing to the cachexia syndrome in which the loss of muscle mass is accelerated (Freeman 2012). This condition is strongly associated with increased mortality in humans (Bielecka‐Dabrowa et al. 2020; Evans et al. 2008; Latenstein et al. 2020). Dogs with cardiac cachexia, which has a similar mechanism, show lower immune response, haematocrit, and haemoglobin and albumin blood concentrations (Freeman 2012; Ineson et al. 2019).

Previous studies (Parker and Freeman 2011; Pedrinelli et al. 2020; Rudinsky et al. 2018) reported that dogs diagnosed with CKD and higher BCS have a higher SR when compared to dogs with ideal and lower BCS. In contrast, the present study showed the link between lower MMS and higher risk of mortality, while BCS had no effect on survival, which contradicts the obesity paradox.

The apparent protective effect of higher BCS observed in previous studies may be confounded by MMS. It is worth noting that weight gain and the development of obesity are the result of increases in both lean and fat masses, with approximately 3/4 (75%) of the added weight coming from adipocyte expansion and the remaining 1/4 (25%) of increased lean body mass (Weeth 2016). Therefore, it is possible that obese animals have greater reserves of lean body mass and, during periods of poor food intake, this could prevent cachexia, rather than the excess body fat itself being beneficial (Weeth 2016).

This study highlights the importance of preserving MMS in the therapeutic management of CKD, which can be aided by a proper dietary approach (Parker 2021). Muscle mass loss in healthy animals is associated with inadequate protein intake, while muscle gain is associated with higher protein intake, a controversial topic for animals diagnosed with CKD. High protein intake has been suggested to exacerbate azotaemia and morbidity in dogs with CKD (Polzin et al. 1983); however, some studies have reported that protein restriction does not alleviate glomerular hypertension, hypertrophy, hyperfiltration, or CKD progression in dogs with induced renal failure (Brown et al. 1990, 1991). More recent evidence in humans and dogs does not support a benefit of protein restriction in CKD, provided that phosphorus intake is restricted, and especially if proteinuria is not present (Bilancio et al. 2019; Bovée 1991; Finco et al. 1992; Sanderson 2018; Shinaberger et al. 2008). When protein restriction is needed, preservation of muscle mass may be aided by using proteins with better digestibility and increased concentration of the branched‐chain amino acids, leucine, isoleucine, and valine. These amino acids have an anticatabolic effect, and their supplementation can promote an increase in body weight and serum albumin concentration in dogs (Zatelli et al. 2017) and cats (Hall et al. 2019).

Our data showed that low MMS is directly associated with lower SR in dogs with CKD rather than BCS. Thus, our study supports that for CKD in dogs, one of the main dietary managements of these patients is to preserve the MMS.

Some limitations of this study are the retrospective approach and the assessment of BCS and MMS using scoring tools, which is a partially subjective method, particularly considering that multiple practitioners contributed to score determination. In overweight and obese dogs, muscle loss might be harder to identify by palpation and inspection, and MMS might be overestimated. Another limitation is the low number of dogs within each subset of BCS and MMS. However, the results are novel and open a new perspective to the obesity paradox discussion and suggest future hypotheses regarding BCS, MMS, and SR in patients with chronic diseases in veterinary medicine.

## Ethics Statement

This study was approved by the Animal Use Ethics Committee from the School of Veterinary and Animal Science of the University of São Paulo (FMVZ/USP), protocol number 3138/2013. The authors confirm that the ethical policies of the journal, as noted on the journal's author guidelines page, have been adhered to, and the appropriate ethical review committee approval has been received. The authors confirm that they have followed EU standards for the protection of animals used for scientific purposes.

## Conflicts of Interest

The authors declare no conflicts of interest.

## References

[jpn70061-bib-0001] Bagheri, M. , J. R. Speakman , S. Shabbidar , F. Kazemi , and K. Djafarian . 2015. “A Dose‐Response Meta‐Analysis of the Impact of Body Mass Index on Stroke and All‐Cause Mortality in Stroke Patients: A Paradox Within a Paradox.” Obesity Reviews 16, no. 5: 416–423. 10.1111/obr.12272. 25753533

[jpn70061-bib-0002] Bagheri, M. , J. R. Speakman , F. Shemirani , and K. Djafarian . 2016. “Renal Cell Carcinoma Survival and Body Mass Index: A Dose–Response Meta‐Analysis Reveals Another Potential Paradox Within a Paradox.” International Journal of Obesity 40, no. 12: 1817–1822. 10.1038/ijo.2016.171. 27686524

[jpn70061-bib-0003] Bailey, J. L. , X. Wang , B. K. England , S. R. Price , X. Ding , and W. E. Mitch . 1996. “The Acidosis of Chronic Renal Failure Activates Muscle Proteolysis in Rats by Augmenting Transcription of Genes Encoding Proteins of the ATP‐Dependent Ubiquitin‐Proteasome Pathway.” Journal of Clinical Investigation 97, no. 6: 1447–1453. 10.1172/JCI118566. 8617877 PMC507204

[jpn70061-bib-0004] Bartges, J. W. 2012. “Chronic Kidney Disease in Dogs and Cats.” Veterinary Clinics of North America: Small Animal Practice 42, no. 4: 669–692. 10.1016/j.cvsm.2012.04.008. 22720808

[jpn70061-bib-0005] Bastien, B. C. , A. Patil , and E. Satyaraj . 2015. “The Impact of Weight Loss on Circulating Cytokines in Beagle Dogs.” Veterinary Immunology and Immunopathology 163, no. 3–4: 174–182. 10.1016/j.vetimm.2014.12.003. 25576490

[jpn70061-bib-0006] Bielecka‐Dabrowa, A. , N. Ebner , M. R. Dos Santos , J. Ishida , G. Hasenfuss , and S. Von Haehling . 2020. “Cachexia, Muscle Wasting, and Frailty in Cardiovascular Disease.” European Journal of Heart Failure 22, no. 12: 2314–2326. 10.1002/ejhf.2011. 32949422

[jpn70061-bib-0007] Bilancio, G. , P. Cavallo , C. Ciacci , and M. Cirillo . 2019. “Dietary Protein, Kidney Function and Mortality: Review of the Evidence From Epidemiological Studies.” Nutrients 11, no. 1: 196. 10.3390/nu11010196. 30669401 PMC6356875

[jpn70061-bib-0008] Bovée, K. C. 1991. “Influence of Dietary Protein on Renal Function in Dogs.” Journal of Nutrition 121: S128–S139. 10.1093/jn/121.suppl_11.S128. 1941208

[jpn70061-bib-0009] Brown, S. A. , D. R. Finco , W. A. Crowell , D. C. Choat , and L. G. Navar . 1990. “Single‐Nephron Adaptations to Partial Renal Ablation in the Dog.” American Journal of Physiology‐Renal Physiology 258, no. 3: F495–F503. 10.1152/ajprenal.1990.258.3.F495. 2316661

[jpn70061-bib-0010] Brown, S. A. , D. R. Finco , W. A. Crowell , and L. G. Navar . 1991. “Dietary Protein Intake and the Glomerular Adaptations to Partial Nephrectomy in Dogs.” Journal of Nutrition 121: S125–S127. 10.1093/jn/121.suppl_11.S125. 1941207

[jpn70061-bib-0011] Brunetto, M. A. , M. O. S. Gomes , M. R. Andre , et al. 2010. “Effects of Nutritional Support on Hospital Outcome in Dogs and Cats.” Journal of Veterinary Emergency and Critical Care 20, no. 2: 224–231. 10.1111/j.1476-4431.2009.00507.x. 20487250

[jpn70061-bib-0012] Carnethon, M. R. , P. J. D. De Chavez , M. L. Biggs , et al. 2012. “Association of Weight Status With Mortality in Adults With Incident Diabetes.” Journal of the American Medical Association 308, no. 6: 581. 10.1001/jama.2012.9282. 22871870 PMC3467944

[jpn70061-bib-0023] Correia, M. T. D. , and D. L. Waitzberg . 2003. “The Impact of Malnutrition on Morbidity, Mortality, Length of Hospital Stay and Costs Evaluated Through a Multivariate Model Analysis.” Clinical Nutrition 22, no. 3: 235–239. 10.1016/S0261-5614(02)00215-7. 12765661

[jpn70061-bib-0013] Evans, W. J. , J. E. Morley , J. Argilés , et al. 2008. “Cachexia: A New Definition.” Clinical Nutrition 27, no. 6: 793–799. 10.1016/j.clnu.2008.06.013. 18718696

[jpn70061-bib-0014] Finco, D. R. , S. A. Brown , W. A. Crowell , C. A. Groves , J. R. Duncan , and J. A. Barsanti . 1992. “Effects of Phosphorus/Calcium‐Restricted and Phosphorus/Calcium‐Replete 32% Protein Diets in Dogs With Chronic Renal Failure.” American Journal of Veterinary Research 53, no. 1: 157–163. 1539911

[jpn70061-bib-0015] Freeman, L. M. 2012. “Cachexia and Sarcopenia: Emerging Syndromes of Importance in Dogs and Cats.” Journal of Veterinary Internal Medicine 26, no. 1: 3–17. 10.1111/j.1939-1676.2011.00838.x. 22111652

[jpn70061-bib-0016] German, A. J. , V. H. Ryan , A. C. German , I. S. Wood , and P. Trayhurn . 2010. “Obesity, Its Associated Disorders and the Role of Inflammatory Adipokines in Companion Animals.” Veterinary Journal 185, no. 1: 4–9. 10.1016/j.tvjl.2010.04.004. 20472476

[jpn70061-bib-0017] Gruberg, L. , N. J. Weissman , R. Waksman , et al. 2002. “The Impact of Obesity on the Short‐Term Andlong‐Term Outcomes After Percutaneous Coronary Intervention: The Obesity Paradox?” Journal of the American College of Cardiology 39, no. 4: 578–584. 10.1016/S0735-1097(01)01802-2. 11849854

[jpn70061-bib-0018] Gupta, T. , D. Kolte , D. Mohananey , et al. 2016. “Relation of Obesity to Survival After In‐Hospital Cardiac Arrest.” American Journal of Cardiology 118, no. 5: 662–667. 10.1016/j.amjcard.2016.06.019. 27381664

[jpn70061-bib-0019] Hall, J. A. , D. A. Fritsch , D. E. Jewell , P. A. Burris , and K. L. Gross . 2019. “Cats With IRIS Stage 1 and 2 Chronic Kidney Disease Maintain Body Weight and Lean Muscle Mass When Fed Food Having Increased Caloric Density, and Enhanced Concentrations of Carnitine and Essential Amino Acids.” Veterinary Record 184, no. 6: 190. 10.1136/vr.104865. 30514741 PMC6589452

[jpn70061-bib-0020] Horwich, T. B. , G. C. Fonarow , M. A. Hamilton , W. R. MacLellan , M. A. Woo , and J. H. Tillisch . 2001. “The Relationship Between Obesity and Mortality in Patients With Heart Failure.” Journal of the American College of Cardiology 38, no. 3: 789–795. 10.1016/S0735-1097(01)01448-6. 11527635

[jpn70061-bib-0021] Ineson, D. L. , L. M. Freeman , and J. E. Rush . 2019. “Clinical and Laboratory Findings and Survival Time Associated With Cardiac Cachexia in Dogs With Congestive Heart Failure.” Journal of Veterinary Internal Medicine 33, no. 5: 1902–1908. 10.1111/jvim.15566. 31317600 PMC6766489

[jpn70061-bib-0022] International Renal Interest Society . 2019. IRIS Staging of *CKD*.

[jpn70061-bib-0024] Juszczyk, K. , S. Kang , S. Putnis , et al. 2020. “High Body Mass Index Is Associated With an Increased Overall Survival in Rectal Cancer.” Journal of Gastrointestinal Oncology 11, no. 4: 626–632. 10.21037/jgo-20-48. 32953146 PMC7475323

[jpn70061-bib-0025] Kumakura, H. , H. Kanai , M. Aizaki , et al. 2010. “The Influence of the Obesity Paradox and Chronic Kidney Disease on Long‐Term Survival in a Japanese Cohort With Peripheral Arterial Disease.” Journal of Vascular Surgery 52, no. 1: 110–117. 10.1016/j.jvs.2010.02.008. 20478682

[jpn70061-bib-0026] Laflamme, D. 1997. “Development and Validation of a Body Condition Score System for Dogs.” Canine Practice 22, no. 4: 10–15.

[jpn70061-bib-0027] Latenstein, A. E. J. , W. P. M. Dijksterhuis , and T. M. Mackay , and Dutch Pancreatic Cancer Group . 2020. “Cachexia, Dietetic Consultation, and Survival in Patients With Pancreatic and Periampullary Cancer: A Multicenter Cohort Study.” Cancer Medicine 9, no. 24: 9385–9395. 10.1002/cam4.3556. 33107709 PMC7774726

[jpn70061-bib-0028] Lennon, H. , M. Sperrin , E. Badrick , and A. G. Renehan . 2016. “The Obesity Paradox in Cancer: A Review.” Current Oncology Reports 18, no. 9: 56. 10.1007/s11912-016-0539-4. 27475805 PMC4967417

[jpn70061-bib-0029] Liu, D. T. , D. C. Brown , and D. C. Silverstein . 2012. “Early Nutritional Support Is Associated With Decreased Length of Hospitalization in Dogs With Septic Peritonitis: A Retrospective Study of 45 Cases (2000–2009).” Journal of Veterinary Emergency and Critical Care 22, no. 4: 453–459. 10.1111/j.1476-4431.2012.00771.x. 22928749

[jpn70061-bib-0030] Michel, K. E. , W. Anderson , C. Cupp , and D. P. Laflamme . 2011. “Correlation of a Feline Muscle Mass Score With Body Composition Determined by Dual‐Energy X‐Ray Absorptiometry.” Supplement, British Journal of Nutrition 106, no. S1: S57–S59. 10.1017/S000711451100050X. 22005437

[jpn70061-bib-0031] Molina, J. , M. Hervera , E. G. Manzanilla , C. Torrente , and C. Villaverde . 2018. “Evaluation of the Prevalence and Risk Factors for Undernutrition in Hospitalized Dogs.” Frontiers in Veterinary Science 5: 205. 10.3389/fvets.2018.00205. 30211177 PMC6123354

[jpn70061-bib-0032] Nentwig, A. , A. Schweighauser , C. Maissen‐Villiger , et al. 2016. “Assessment of the Expression of Biomarkers of Uremic Inflammation in Dogs With Renal Disease.” American Journal of Veterinary Research 77, no. 2: 218–224. 10.2460/ajvr.77.2.218. 27027717

[jpn70061-bib-0033] Niedziela, J. , B. Hudzik , N. Niedziela , et al. 2014. “The Obesity Paradox in Acute Coronary Syndrome: A Meta‐Analysis.” European Journal of Epidemiology 29, no. 11: 801–812. 10.1007/s10654-014-9961-9. 25354991 PMC4220102

[jpn70061-bib-0034] Okada, Y. , M. Kobayashi , M. Sawamura , and T. Arai . 2017. “Comparison of Visceral Fat Accumulation and Metabolome Markers Among Cats of Varying BCS and Novel Classification of Feline Obesity and Metabolic Syndrome.” Frontiers in Veterinary Science 4: 17. 10.3389/fvets.2017.00017. 28261588 PMC5306360

[jpn70061-bib-0035] Park, J. , S.‐F. Ahmadi , E. Streja , et al. 2014. “Obesity Paradox in End‐Stage Kidney Disease Patients.” Progress in Cardiovascular Diseases 56, no. 4: 415–425. 10.1016/j.pcad.2013.10.005. 24438733 PMC4733536

[jpn70061-bib-0036] Parker, V. J. 2021. “Nutritional Management for Dogs and Cats With Chronic Kidney Disease.” Veterinary Clinics of North America: Small Animal Practice 51, no. 3: 685–710. 10.1016/j.cvsm.2021.01.007. 33773648

[jpn70061-bib-0037] Parker, V. J. , and L. M. Freeman . 2011. “Association Between Body Condition and Survival in Dogs With Acquired Chronic Kidney Disease.” Journal of Veterinary Internal Medicine 25, no. 6: 1306–1311. 10.1111/j.1939-1676.2011.00805.x. 22092621

[jpn70061-bib-0038] Pawelec, G. , D. Goldeck , and E. Derhovanessian . 2014. “Inflammation, Ageing and Chronic Disease.” Current Opinion in Immunology 29: 23–28. 10.1016/j.coi.2014.03.007. 24762450

[jpn70061-bib-0039] Pedrinelli, V. , D. M. Lima , C. N. Duarte , et al. 2020. “Nutritional and Laboratory Parameters Affect the Survival of Dogs With Chronic Kidney Disease.” PLoS One 15, no. 6: e0234712. 10.1371/journal.pone.0234712. 32603378 PMC7326232

[jpn70061-bib-0040] Pegram, C. , C. Gray , R. M. A. Packer , et al. 2021. “Proportion and Risk Factors for Death by Euthanasia in Dogs in the UK.” Scientific Reports 11, no. 1: 9145. 10.1038/s41598-021-88342-0. 33947877 PMC8096845

[jpn70061-bib-0041] Pelander, L. , J. Häggström , A. Larsson , et al. 2019. “Comparison of the Diagnostic Value of Symmetric Dimethylarginine, Cystatin C, and Creatinine for Detection of Decreased Glomerular Filtration Rate in Dogs.” Journal of Veterinary Internal Medicine 33, no. 2: 630–639. 10.1111/jvim.15445. 30791142 PMC6430914

[jpn70061-bib-0042] Pirlich, M. , T. Schütz , K. Norman , et al. 2006. “The German Hospital Malnutrition Study.” Clinical Nutrition 25, no. 4: 563–572. 10.1016/j.clnu.2006.03.005. 16698132

[jpn70061-bib-0043] Polzin, D. J. 2011a. “Chronic Kidney Disease.” In Nephrology and Urology of Small Animals, edited by Em. J. Bartges and D. J. Polzin, Org. , 431–471. Wiley. 10.1002/9781118785546.ch48.

[jpn70061-bib-0044] Polzin, D. J. 2011b. “Chronic Kidney Disease in Small Animals.” Veterinary Clinics of North America: Small Animal Practice 41, no. 1: 15–30. 10.1016/j.cvsm.2010.09.004. 21251509

[jpn70061-bib-0045] Polzin, D. J. , C. A. Osborne , J. B. Stevens , and D. W. Hayden . 1983. “Influence of Modified Protein Diets on the Nutritional Status of Dogs With Induced Chronic Renal Failure.” American Journal of Veterinary Research 44, no. 9: 1694–1702. 6625324

[jpn70061-bib-0046] Romano, F. R. , C. R. Heinze , L. G. Barber , J. B. Mason , and L. M. Freeman . 2016. “Association Between Body Condition Score and Cancer Prognosis in Dogs With Lymphoma and Osteosarcoma.” Journal of Veterinary Internal Medicine 30, no. 4: 1179–1186. 10.1111/jvim.13965. 27279003 PMC5153966

[jpn70061-bib-0047] Romero‐Corral, A. , V. M. Montori , V. K. Somers , et al. 2006. “Association of Bodyweight With Total Mortality and With Cardiovascular Events in Coronary Artery Disease: A Systematic Review of Cohort Studies.” Lancet 368, no. 9536: 666–678. 10.1016/S0140-6736(06)69251-9. 16920472

[jpn70061-bib-0048] Roura, X. 2019. Risk Factors in Dogs and Cats for Development of Chronic Kidney Disease. International Renal Interest Society. https://www.iris‐kidney.com/ckd‐risk‐factors.

[jpn70061-bib-0049] Rudinsky, A. J. , L. M. Harjes , J. Byron , et al. 2018. “Factors Associated With Survival in Dogs With Chronic Kidney Disease.” Journal of Veterinary Internal Medicine 32, no. 6: 1977–1982. 10.1111/jvim.15322. 30325060 PMC6271312

[jpn70061-bib-0050] Sanderson, S. L. 2018, maio 3. Rethinking Protein Restriction in Aging Dogs and Cats With Chronic Kidney Disease. Companion Animal Nutrition Summit.

[jpn70061-bib-0051] Shinaberger, C. S. , S. Greenland , J. D. Kopple , et al. 2008. “Is Controlling Phosphorus by Decreasing Dietary Protein Intake Beneficial or Harmful in Persons With Chronic Kidney Disease?” American Journal of Clinical Nutrition 88, no. 6: 1511–1518. 10.3945/ajcn.2008.26665. 19064510 PMC5500249

[jpn70061-bib-0052] Slupe, J. L. , L. M. Freeman , and J. E. Rush . 2008. “Association of Body Weight and Body Condition With Survival in Dogs With Heart Failure.” Journal of Veterinary Internal Medicine 22, no. 3: 561–565. 10.1111/j.1939-1676.2008.0071.x. 18466257

[jpn70061-bib-0053] Tan, X.‐F. , J.‐X. Shi , and M. H. Chen . 2016. “Prolonged and Intensive Medication Use Are Associated With the Obesity Paradox After Percutaneous Coronary Intervention: A Systematic Review and Meta‐Analysis of 12 Studies.” BMC Cardiovascular Disorders 16, no. 1: 125. 10.1186/s12872-016-0310-7. 27267233 PMC4895875

[jpn70061-bib-0054] Tvarijonaviciute, A. , F. Tecles , S. Martínez‐Subiela , and J. J. Cerón . 2012. “Effect of Weight Loss on Inflammatory Biomarkers in Obese Dogs.” Veterinary Journal 193, no. 2: 570–572. 10.1016/j.tvjl.2012.02.015. 22464400

[jpn70061-bib-0055] Vashistha, T. , R. Mehrotra , J. Park , et al. 2014. “Effect of Age and Dialysis Vintage on Obesity Paradox in Long‐Term Hemodialysis Patients.” American Journal of Kidney Diseases 63, no. 4: 612–622. 10.1053/j.ajkd.2013.07.021. 24120224 PMC3969454

[jpn70061-bib-0056] Vendramini, T. H. A. , H. T. Macedo , A. R. Amaral , et al. 2020. “Gene Expression of the Immunoinflammatory and Immunological Status of Obese Dogs before and After Weight Loss.” PLoS One 15, no. 9: e0238638. 10.1371/journal.pone.0238638. 32966299 PMC7510989

[jpn70061-bib-0057] Vertloo, L. V. 2025. Renal Dysfunction in Dogs and Cats (V. 2025). Merck Veterinary Manual. https://www.merckvetmanual.com/urinary‐system/noninfectious‐diseases‐of‐the‐urinary‐system‐in‐small‐animals/renal‐dysfunction‐in‐dogs‐and‐cats.

[jpn70061-bib-0058] Von Haehling, S. , M. Lainscak , J. Springer , and S. D. Anker . 2009. “Cardiac Cachexia: A Systematic Overview.” Pharmacology & Therapeutics 121, no. 3: 227–252. 10.1016/j.pharmthera.2008.09.009. 19061914

[jpn70061-bib-0059] Weeth, L. P. 2016. “Other Risks/Possible Benefits of Obesity.” Veterinary Clinics of North America: Small Animal Practice 46, no. 5: 843–853. 10.1016/j.cvsm.2016.04.007. 27267439

[jpn70061-bib-0060] Yamauchi, Y. , W. Hasegawa , H. Yasunaga , et al. 2014. “Paradoxical Association Between Body Mass Index and In‐Hospital Mortality in Elderly Patients With Chronic Obstructive Pulmonary Disease in Japan.” International Journal of Chronic Obstructive Pulmonary Disease 9: 1337. 10.2147/COPD.S75175. 25525351 PMC4266240

[jpn70061-bib-0061] Zapata, R. C. , M. D. Meachem , N. C. Cardoso , et al. 2017. “Differential Circulating Concentrations of Adipokines, Glucagon and Adropin in a Clinical Population of Lean, Overweight and Diabetic Cats.” BMC Veterinary Research 13, no. 1: 85. 10.1186/s12917-017-1011-x. 28376869 PMC5379571

[jpn70061-bib-0062] Zatelli, A. , P. D'ippolito , X. Roura , and E. Zini . 2017. “Short‐Term Effects of Dietary Supplementation With Amino Acids in Dogs With Proteinuric Chronic Kidney Disease.” Canadian Veterinary Journal = La Revue Veterinaire Canadienne 58, no. 12: 1287–1293. 29203938 PMC5680804

